# The Impact of Parental Death in Childhood on Sons’ and Daughters’ Status Attainment in Young Adulthood in the Netherlands, 1850–1952

**DOI:** 10.1007/s13524-019-00808-z

**Published:** 2019-08-16

**Authors:** Matthias Rosenbaum-Feldbrügge

**Affiliations:** Radboud Group for Historical Demography and Family History, Department of History, Radboud University, Erasmusplein 1, 6525HT Nijmegen, The Netherlands

**Keywords:** Parental death, Status attainment, Labor market transformation, Social mobility, Paternal absence

## Abstract

Previous research on the impact of parental loss on labor market outcomes in adulthood has often suffered from low sample sizes. To generate further insights into the long-term consequences of parental death, I use the Historical Sample of the Netherlands (HSN). The HSN contains occupational information on life courses of a sample of more than 8,000 males and almost 7,000 females born between 1850 and 1922, a period of important labor market transformations. Roughly 20 % of the sample population experienced parental death before age 16. Linear regression models show that maternal loss is significantly associated with lower occupational position in adulthood for both men and women, which points to the crucial importance of maternal care in childhood for socioeconomic outcomes in later life. This interpretation is supported by the finding that a stepmother’s entry into the family is positively related with sons’ occupational position later in life. In contrast to expectations, the loss of economic resources related to the father’s death is generally not associated with lower status attainment in adulthood for men or for women. The results indicate, however, that the negative consequences of paternal death on men’s socioeconomic outcomes decreased over time, illustrating the complex interaction between individual life courses and surrounding labor market transformations.

**Electronic supplementary material:**

The online version of this article (10.1007/s13524-019-00808-z) contains supplementary material, which is available to authorized users.

## Introduction

Parental death is one of the most traumatic events that can occur in childhood and may influence an affected individual’s life course in many ways. Compared with children who grow up with both biological parents, parentally bereaved children have significantly lower self-esteem and experience more feelings of helplessness, sadness, guilt, and anger (Worden 1996; Worden and Silverman 1996). Parental loss during childhood is also associated with weaker educational attainment and greater risk of school failure during adolescence (Abdelnoor and Hollins 2004; Amato and Anthony 2014; Amato and Keith 1991; Berg et al. 2014; Steele et al. 2009), which might be explained by bereaved children’s higher chances of developing mental health problems (Björkenstam et al. 2016; Veldman et al. 2015). Indeed, bereaved adolescents in contemporary Western societies are more likely to report various behavioral problems, to attempt suicide, to be hospitalized due to psychiatric disorders, and to commit violent criminal acts (Feigelman et al. 2017; Jakobsen and Christiansen 2011; Wilcox et al. 2010).

Recent research has suggested that the negative health consequences of parental mortality fade once bereaved children enter adulthood (Feigelman et al. 2017). Support for this theory is mixed. On the one hand, no long-term relationship has been found between early parental death and adult depression, suicidality, use of drugs, criminal involvements, and use of mental health services (Feigelman et al. 2017; Kessler et al. 1997; Stikkelbroek et al. 2012). On the other hand, studies have discovered that parentally bereaved adults have a greater risk of depression, hospitalization due to affective disorders (Appel et al. 2013; Berg et al. 2016; Mack 2001), suicide attempts (Hollingshaus and Smith 2015; Rostila et al. 2016), and premature death (Li et al. 2014). Mixed results have also been found in research on the long-term mortality consequences of parental loss in childhood. Whereas some studies discovered that individuals experiencing parental mortality during infancy and childhood have lower survival chances in later life (Campbell and Lee 2009; Smith et al. 2014), others found higher survival chances (Smith et al. 2009; van Poppel and Liefbroer 2005) or hardly any mortality consequences (Gagnon and Mazan 2009; Todd et al. 2017; Willführ 2009).

Even though parental loss is associated with lower educational attainment in adolescence, the existing literature is inconsistent with regard to the long-term effect of parental loss on various labor market outcomes in adulthood. For example, research has shown that bereaved individuals have a higher risk of unemployment and are more likely to report difficulties at work (Brent et al. 2012; Corak 2001; Feigelman et al. 2017; Fronstin et al. 2001; Parsons 2011). Conversely, several studies have suggested that parental loss in childhood is not negatively associated with earnings and occupational status attainment in adulthood (Biblarz and Gottainer 2000; Corak 2001; Lang and Zagorsky 2001). These considerable inconsistencies in studies on long-term effects of early parental death on health and labor market outcomes have led researchers to suggest that moderating factors, such as the child’s age at bereavement and the sex of the deceased parent, play an important role in explaining this relationship (Luecken 2008).

The present article contributes to the literature on long-term consequences of parental death in childhood by investigating its impact on sons’ and daughters’ status attainment in a historical population. Moreover, the large data set used here also sheds light on the role of moderating factors. The article examines two research questions in particular. First, does the proposed negative effect of parental death during childhood and adolescence persist after entry into adulthood to affect status attainment? Second, do moderating factors—such as sex of the deceased parent, child’s age at parental death, presence of stepparents, and period of birth—attenuate the potentially negative effect of parental death?

I use the Historical Sample of the Netherlands (HSN) to examine these research questions. The HSN follows the life courses of approximately 37,000 males and females born in the Netherlands between 1850 and 1922. Using historical data has several advantages compared with data on present-day populations. First, studies on contemporary Western countries often suffer from small numbers of parentally bereaved children and therefore do not differentiate between paternal and maternal death or ages at bereavement (e.g., Amato and Anthony 2014; Feigelman et al. 2017; Parsons 2011). People born in the Netherlands in the period of consideration, however, were much more likely to experience parental death during childhood, which enables me to test specific hypotheses about subgroups. Second, the HSN covers an entire century, which makes it possible to follow thousands of individuals over a period when important societal, cultural, and economic developments took place. Finally, vulnerable individuals in the sample were, in relative terms, insufficiently supported by poverty relief. Studying this period is therefore useful to reveal the potentially damaging long-term effects of childhood vulnerability prior to the development of modern welfare states.

## Theoretical Background

Losing a parent during childhood was a common experience for children born in the Netherlands in the second half of the nineteenth and the beginning of the twentieth century. In a recent article, van Poppel et al. (2013) estimated the percentage of Dutch children that had experienced parental mortality in the last 150 years. For example, more than 10 % of children born between 1850 and 1879 had lost a mother and/or father at the age of 7, and nearly 25 % had experienced parental loss by the age of 15. The share of parentally bereaved children decreased due to lower parental mortality risk, which declined especially from 1880 onward (Wolleswinkel-van den Bosch et al. 1998). Nevertheless, nearly 6 % of children born between 1900 and 1922 faced parental loss by age 7, and more than 13 % experienced parental loss by age 15. For comparison, in the latest birth cohorts for which information is available—1975 to 1985—these figures are 1 % and 3 %, respectively.

In an earlier contribution, van Poppel et al. (1998) investigated whether paternal death before marriage affects a son’s occupational status in adulthood. They discovered that in The Hague in the nineteenth century, paternal death before a son’s marriage was associated with lower occupational position. This effect was strongest for sons who had lost their father before age 15. According to the authors, this is mainly attributable to the loss of paternal investment and professional networks. This article expands the study of the effects of parental death on children’s status attainment in several important ways. Among other things, I follow many more research persons born all over the Netherlands, examine the marriage mobility of parentally bereaved daughters by comparing their husband’s and father’s occupational position, and also consider the effect of maternal death. Finally, this study exploits occupational entries not only from marriage certificates but also from population registers to include never-married sons, who might differ from their married counterparts (Delger and Kok 1998).

**Fig. 1:**
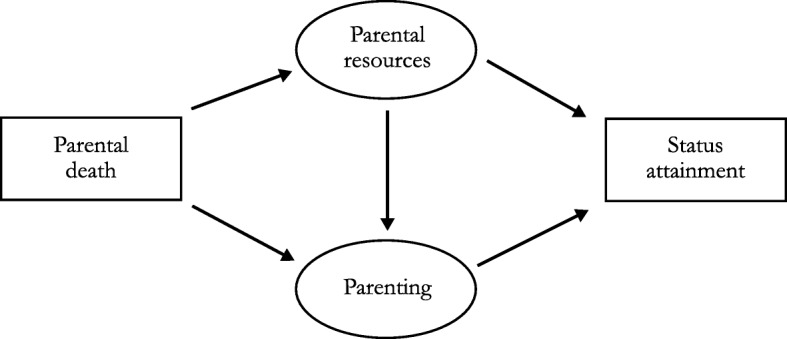
Simplified pathway between parental death in childhood and status attainment in adulthood

The theoretical considerations here are based on a framework described by McLanahan and Percheski (2008). A slightly modified version of their theoretical framework is depicted in Fig. 1, which shows the simplified pathway between parental death in childhood and status attainment in adulthood. As shown in the figure, the impact of parental death on status attainment operates through the two mechanisms of parental resources and parenting quality. Parental resources (e.g., income), the first pathway, are important for a child’s well-being and future status attainment (Blau and Duncan 1967; Duncan et al. 2012; Meyer 1997). The loss of a parent, however, deprives the child of assets that would have benefitted his or her career in later life. Especially in the past, parental death often meant that the household’s risk of poverty increased and that children’s plans for education or work were thwarted (Bras and Kok 2004). Therefore, parental mortality is expected to reduce parental resources, thereby having a harmful long-term impact on status attainment.

The second pathway operates through parenting quality. Parental loss is related to higher levels of stress and higher risk of mental health problems in the surviving parent (Worden 1996), which might affect parenting quality negatively. Lower levels of the surviving parent’s interaction with the child, in turn, are associated with economic adversity in later life (Berg et al. 2017). Parental mental health problems and lower parenting quality can also be caused by financial insecurity. Accordingly, parenting quality is also directly affected by losing parental resources, such as income (McLanahan and Percheski 2008). Taken together, a detrimental relationship between parental loss during childhood and occupational attainment in later life is expected, which operates through parental resources and parenting quality. Moreover, I hypothesize that this negative effect does not fade after the presence of stepparents and female kin as well as several adulthood variables, such as internal migration, are controlled for.

- *Hypothesis 1a (H1a):* Parental death in childhood is generally associated with lower status attainment in adulthood.

- *Hypothesis 1b (H1b):* The negative impact of parental death on status attainment persists after parental remarriage, the presence of female kin, and adulthood variables are controlled for.

During the period of consideration, Dutch society was characterized by a strict division of tasks between husband and wife (Pfau-Effinger 2004; Pott-Buter 1993; Schmidt and van Nederveen Meerkerk 2012). The husband was mainly responsible for earning money, and the wife usually was in charge of unpaid domestic labor and childcare. Accordingly, the mother was the main source of the child’s emotional support, whereas the father was responsible for the material well-being of his children. Although widowed mothers with small children were a favorite target group of locally organized charity institutions funded by municipalities, churches, and private foundations (Schmidt 2007; van Leeuwen 1998; van Loo 1981; van Poppel 1992), widows from the lower social classes in particular struggled with generating income, and their families were more likely to experience impoverishment (Schmidt 2007).^1^ Therefore, I expect that paternal death mainly operates through losing economic resources and higher risk of poverty and malnutrition (Derosas and Oris 2002). Maternal death, however, mainly operates through a decline in parenting quality and emotional support (Sear and Mace 2008) and higher risk of family dissolution (Rosenbaum-Feldbrügge 2018). As a consequence, I hypothesize that maternal death and paternal death are equally detrimental when it comes to status attainment in young adulthood, but for different reasons.

- *Hypothesis 2 (H2):* Maternal loss and paternal loss are equally harmful to children’s socioeconomic positions in adulthood.

Research has suggested that a mother’s care is of prime importance in the child’s first years of life and that maternal death dramatically increases infant and childhood mortality rates in historical as well as low-income populations (for an overview, see Sear and Mace 2008). Because other family members, such as sisters or other female kin, usually cannot fully compensate for loss of maternal care (Sear and Coall 2011), I expect that maternal loss in early childhood scars the child over the entire life course. The negative effect of maternal loss is expected to lessen with children’s increased ages at loss, when maternal care and parenting become relatively less important. Given that exposure to adverse conditions during the first years of life has a stronger negative impact on later-life outcomes than during late childhood and adolescence (Brooks-Gunn and Duncan 1997; Duncan et al. 2012), I expect that paternal death and its negative consequences are also specifically harmful in early life. Based on these considerations, I derive the third hypothesis:

- *Hypothesis 3 (H3):* Parental death in infancy and childhood is more harmful to status attainment than parental death in adolescence.

Remarriage is a strategy for the surviving parent to cope with the loss of a spouse. From an economic standpoint, the stepparent may reduce the negative impact of parental loss by contributing to the family’s income or by satisfying the household’s need for a caregiver. Accordingly, the entry of a stepparent in nineteenth century Netherlands is associated with a lower risk of family dissolution (Rosenbaum-Feldbrügge 2018). Therefore, I expect that the entry of a stepparent reduces the negative impact of parental death in childhood.

- *Hypothesis 4 (H4):* Parental remarriage reduces the negative relationship between parental death and status attainment in later life.

In the second half of the nineteenth and the beginning of the twentieth century, the Netherlands experienced tremendous economic and social developments. The period was characterized by accelerating modernization, industrialization, urbanization, technological developments, and rising living standards (van Zanden and van Riel 2004). Education became much more important. Investments in education increased to a large extent, and compulsory schooling was introduced in 1901, which further increased school participation rates (van der Voort 1994). Social mobility and opportunities for personal advancement increased during the end of the nineteenth and beginning of the twentieth century because of increased demand for skilled and educated workers (van Zanden and van Riel 2004). As a result of modernization, educational expansion, and the structural transformation of the labor market, individual occupational status attainment became less dependent on parental background (Knigge et al. 2014). In line with these considerations, I hypothesize that the negative effect of parental death decreased over time.

- *Hypothesis 5 (H5):* The negative impact of parental death on status attainment becomes weaker over time.

## Data, Variables, and Methods

The 2010.01 release of the Historical Sample of the Netherlands (HSN) follows the life courses of a representative sample of more than 37,000 males and females (hereafter referred to as research persons) born 1850–1922. The sampling strategy ensured that research persons are not clustered within households, which implies that each research person has a unique set of parents. The HSN is based on birth, marriage, and death certificates as well as on population registers introduced throughout the country in 1850. Population registers are a unique data source in that they contain continuously updated information about demographic events of all the household members, such as birth, migration, and death (Mandemakers 2002). Thus, population registers enable the researcher to identify changes in the research persons’ household composition—for example, the death of a parent, presence of female kin, and entry of a stepparent. Occupational information is available on the birth certificates, marriage certificates, and population registers. Moreover, research persons who migrated within the country are followed. Few individuals who migrated to another country, however, are lost from observation. From the end of the 1930s onward, population registers where replaced by personal cards, which do not include the date of occupational entry. Therefore, after 1940, I consult information only from the research person’s marriage certificates. The last occupation was recorded in 1952.

HSN occupations derived from population registers and marriage certificates are coded into the Historical International Standard Classification of Occupations (HISCO). HISCO is a version of three-digit ISCO-68 adapted to historical purposes and is based on occupations derived from parish and civil registration documents from several Western countries covering the nineteenth and the first half of the twentieth century (van Leeuwen et al. 2002). HISCO is translated into both the continuous HISCAM scale and the categorical HISCLASS scheme.

HISCAM is a social stratification scale that estimates the social prestige of occupations. It is continuous, ranges from 39.9 to 99, and is centered on a mean of 50 with a standard deviation of 10. Higher values indicate higher occupational prestige. For instance, lawyers receive the highest possible score of 99, and domestic servants are assigned a score of 39.9 (Lambert et al. 2013). HISCAM will be applied in the descriptive and multivariate analyses.

HISCLASS is a categorical classification scheme containing 12, 7, or 5 hierarchical social classes (van Leeuwen and Maas 2011). Five social classes are used in this article to graphically observe transformations in the structure of the labor market over time: (1) elite, (2) lower middle class (mainly white-collar workers), (3) skilled workers, (4) self-employed farmers and fishermen, and (5) unskilled workers.^2^

Males are included in the study if they had a paternal occupation recorded at birth and an own occupation recorded between ages 23 and 33. This age range is defined as the first 10 years of adulthood because between 1838 and 1905, individuals below age 23 were legally regarded as minors (van Solinge et al. 2000). If several occupations from marriage certificates and the population registers were recorded between ages 23 and 33, the occupation with the highest status was chosen. Likewise, I include females if they had a paternal occupation recorded at birth and their husband had an occupation recorded in young adulthood. Women’s status attainment is measured indirectly by taking the husband’s occupation into account because female occupations were often not registered in historical sources (Janssens 2014; Walhout and van Poppel 2003). Twelve women in the sample were married to a man in the sample, meaning that 12 sons in the male sample are identical to husbands in the female sample. I keep these individuals in both the male and female sample because their total number is negligible.

An overview of the sample selection process and the inclusion criteria is depicted in Fig. A1 in the online appendix. Roughly 12,000 males and females, respectively, had a paternal occupation recorded at birth and survived until age 23. Unfortunately, occupations were not entered regularly on population registers, which means that only 8,187 males and 6,915 husbands of females in the sample had an occupational entry in young adulthood. The numbers for females are lower because around 15 % of the women of marital age remained celibate during the period of consideration (van Poppel 1992:21–22) and had to be excluded. Roughly 20 % of the research persons in the final sample had experienced parental death before the age of 16.

Having lost a parent between birth and age 16 is the main independent variable of interest. The control group contains those males and females who did not experience parental death before age 16. Covariates are divided into childhood, orphanhood, and adulthood variables. Among the childhood variables are the father’s HISCAM score derived from the child’s birth certificate and the number of younger and older brothers and sisters. The number of siblings that died before their 10th birthday is considered in a separate group to control for poor living conditions and shared adversity. Additional childhood variables are the mother’s age at the birth of the research person and the father’s literacy, defined by the father’s ability to sign his son’s birth certificate. Given that the Protestant population was politically and economically more powerful than its Catholic counterpart (Engelen and Kok 2002), I divide religion into four categories: Catholics, orthodox Protestants, liberal Protestants, and unknown/other. The distinction between orthodox Protestants and liberal Protestants is based on the congregation and the number of orthodox and liberal ministers elected by the communities of the Dutch Reformed Church. If only orthodox ministers were present in a municipality, the members of the Dutch Reformed Church were considered orthodox Protestants (Kok 2017). Finally, following van Poppel and colleagues (van Poppel et al. 2013), the period of birth is divided into three subperiods: 1850–1879, 1880–1899, and 1900–1922. These subperiods closely follow the labor market transformations in the Netherlands in the period of consideration.

Orphanhood variables contain the entry of a stepmother or stepfather, presence of female kin (aunts and grandmothers) after parental death, sex of the deceased parent, and age of the child at bereavement. The age of the child at parental death is categorized into four groups: birth to 2 years, early childhood (ages 3 to 6), late childhood (ages 7 to 10), and early adolescence (ages 11 to 15). Adulthood variables are measured at the time the occupation was recorded and include the research person’s migration to another province compared with the birth province, the actual province of residence, whether the person lived in an urban or rural area, and the civil status. Urban communities are defined as having more than 10,000 inhabitants and less than 2.5 % of the population employed in the agricultural sector as per a national census from 1899 (Kooij 1985).

In the multivariate analyses, I apply linear regression status-attainment models (Hendricks and Ganzeboom 1998) and regress the children’s occupational prestige in young adulthood on childhood, orphanhood, and adulthood variables. The dependent variable is son’s and husband’s HISCAM score. Because HISCAM is constructed as a continuous variable, I use ordinary least squares (OLS) regression with robust standard errors.^3^ In the first part of the analysis, I introduce parental death as a dummy variable and develop the models stepwise. The first model controls for the childhood variables, the second model adds stepparents and female kin to the analysis, and the third model controls for the remaining adulthood variables. The second part of the analysis divides parental death by the sex of the deceased parent (first model) and the age of the child at bereavement (second model). The third model introduces interactions with the birth period to test whether the impact of paternal and maternal death changed over time.

## Results

### Labor Market Transformation in the Period of Consideration

**Fig. 2:**
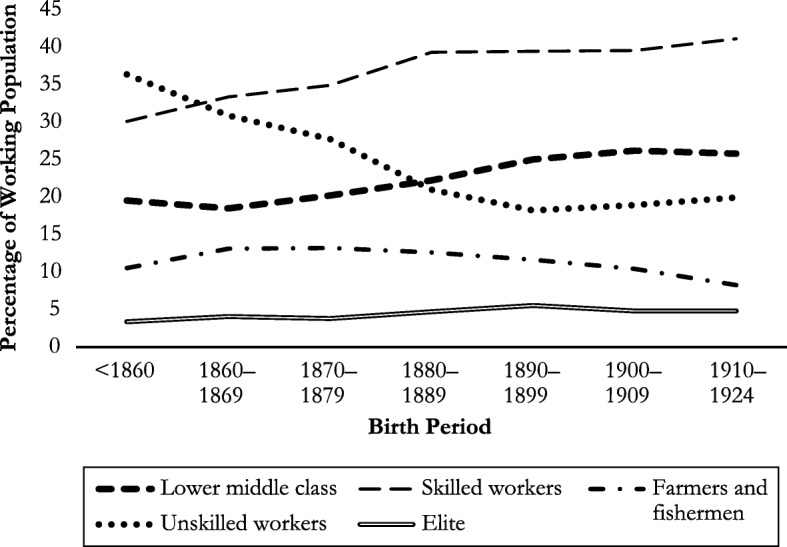
Development of HISCLASS occupational classes over time based on sample males and husbands of sample females (*N* = 15,102), by birth period

Figure 2 depicts changes in the occupational structure over time, based on the HISCLASS classes of the male research persons and the husbands of the female research persons. Among males born before 1860, unskilled workers accounted for the highest share of the work force (35 %), followed by skilled workers (30 %) and members of the lower middle class (20 %). Whereas the percentage of unskilled workers dropped dramatically to below 20 % until the beginning of the twentieth century, increasingly more males born in this period performed skilled work and became members of the lower middle class. The share of self-employed farmers and fishermen decreased slowly but continuously, whereas the share of elite occupations remained more or less stable at a low level. Figure 2 indicates that the Dutch labor market was undergoing structural change at the end of the nineteenth and first half of the twentieth century. Unskilled labor was increasingly replaced by skilled and educated work.

### Descriptive Statistics

Summary statistics offer an initial insight into the impact of parental death during childhood on occupational status in adulthood. Table 1 presents mean summary statistics of the variables used in the analysis by sex and by parental death before age 16. Additionally, *p* values of two-tailed *t* tests for continuous variables and chi-square tests for categorical variables are presented to assess whether parentally bereaved children differed significantly from their nonbereaved counterparts. With regard to childhood conditions, the test statistics reveal important differences. Bereaved children, on average, had older mothers, more older siblings, and fewer younger siblings, and were more likely to be born between 1850 and 1879. Parentally bereaved sons were slightly more likely to experience sibling deaths. Differences with regard to father’s literacy, however, are not observed. The mother’s older age—and hence, the higher number of older siblings—is expected because the mortality risk for older parents is generally higher. The lower number of younger siblings is explained by parental deaths being more frequent toward the end of the parental reproductive age. The high share of orphans in the first period illustrates that parental death indeed decreased over time. Differences are also observed regarding paternal occupational position. Whereas male orphans did not have significantly lower paternal HISCAM scores at birth, parentally bereaved daughters appear to have been slightly disadvantaged at birth compared with nonbereaved daughters (0.43 HISCAM points). Furthermore, the highest paternal HISCAM score ever recorded in the certificates is, on average, significantly lower among orphaned sons and orphaned daughters. I investigate this further later in the article.

**Table 1:** Variable summary statistics, by sex of research person and parental death

	Males			Females		
	No Parental Death Before Age 16	Parental Death Before Age 16	*p* Values of *t* Test or Chi-Square Test^a^	No Parental Death Before Age 16	Parental Death Before Age 16	*p* Values of *t* Test or Chi-Square Test^a^
Childhood Variables						
Father’s HISCAM at birth	52.53	52.19	.123	52.29	51.86	.031
Father’s highest HISCAM	54.78	53.72	.000	54.51	53.46	.000
Siblings						
Number younger brothers	1.4	1.1	.000	1.4	1.1	.000
Number older brothers	1.2	1.5	.000	1.2	1.5	.000
Number younger sisters	1.4	1.1	.000	1.4	1.1	.000
Number older sisters	1.2	1.5	.000	1.1	1.4	.000
Total siblings	5.2	5.2	.982	5.2	5.0	.134
Number of sibling deaths before age 10 (%)						
0	58.45	55.33		58.09	55.71	
1	23.63	24.18		23.33	24.78	
2+	17.91	20.48	.029	18.57	19.51	.282
Birth period (%)						
1850––1879	28.16	38.18		29.84	35.24	
1880––1899	42.94	42.24		41.80	43.10	
1900––1922	28.90	19.58	.000	28.36	21.66	.000
Mother’s age at birth (%)						
<25	14.01	8.97		15.93	11.57	
25––35	61.56	56.18		59.57	54.97	
>35	24.43	34.85	.000	24.50	33.46	.000
Father’s literacy (%)						
Literate	83.78	84.85		81.68	82.94	
Illiterate	6.20	6.00		6.90	7.27	
Unknown	10.02	9.15	.530	11.42	9.79	.220
Religion (%)						
Catholic	32.00	32.97		30.61	34.64	
Liberal protestant	42.99	42.24		44.49	44.07	
Orthodox protestant	14.92	14.79		14.82	13.65	
Unknown/other	10.10	10.00	.901	10.08	7.64	.004
Orphanhood Variables						
Stepparent (%)	––	37.15		––	35.91	.481
Presence of female kin (%)	––	17.39		––	15.06	.086
Age at parental death (%)						
0––2	––	16.36		––	16.32	
3––6	––	25.64		––	24.41	
7––10	––	27.45		––	26.41	
11––15	––	30.55		––	32.86	.570
Sex of deceased parent (%)						
Father	––	47.52		––	48.15	
Mother	––	45.39		––	46.07	
Both	––	7.09		––	5.79	.354
Adulthood Variables						
HISCAM, age 23 to 33	56.24	55.17	.000	––	––	
Husband’s HISCAM, age 23 to 33	––	––		56.11	55.33	.012
Civil status (%)						
Ever married	68.04	63.64		100.00	100.00	
Unmarried	27.81	31.27		––	––	
Unknown	4.15	5.09	.002	––	––	
Migration and place of residence (%)						
No migration/urban	30.60	29.58		32.76	29.75	
No migration/rural	44.24	46.48		44.60	46.22	
Migration/urban	8.95	7.39		8.80	8.53	
Migration/rural	6.15	5.64		5.16	5.27	
Unknown	10.07	10.91	.130	8.68	10.24	.149
Age of male research person	26.60	26.62	.724	––	––	
Age of husband	––	––		26.92	27.02	.284
Total Number	6,537	1,650		5,567	1,348	

^a^Two-tailed *t* tests are applied.

The sample means of the orphanhood variables do not reveal significant contrasts between male and female orphans. Only around 16 % of the half-orphaned children experienced the presence of female kin in the household, consistent with the finding that extended households were not common in Dutch society (Riswick 2018). With regard to occupational status in adulthood, younger male generations generally obtained considerably higher HISCAM scores compared with their fathers and fathers-in-law, which is in line with the findings presented in the previous section. Bereaved sons and the husbands of bereaved daughters, however, scored around 1 HISCAM point lower than the nonbereaved control group. Concerning the other adulthood conditions, bereaved sons were less likely to be married in young adulthood, but no differences are observed regarding internal migration, the place of residence, and the age when the occupations were recorded.

To gain more understanding about the occupational status of fathers and sons/sons-in-law, Table 2 shows mean HISCAM scores by sex of the deceased parent. The table is not adjusted for age at bereavement because this would substantially decrease the number of observations per subgroup. Numbers in parentheses are *p* values of two-sample *t* tests, with nonorphans as the reference group. For both males and females, no structural differences are found with regard to father’s HISCAM score at birth between bereaved and nonbereaved children. Only the fathers of male full orphans and female maternal orphans scored lower at the 10 % level. The family’s social position at birth therefore does not appear to be strongly related with the risk of both maternal and paternal death.^4^

**Table 2:** Mean HISCAM scores by type of parental death and sex

	No Parental Death	Paternal Death	Maternal Death	Both Parents Died
Males^a^				
Father’s HISCAM at birth	52.53	52.24	52.31	51.11
		(.336)	(.482)	(.057)
Father’s highest HISCAM	54.78	53.38	54.31	52.20
		(.000)	(.176)	(.002)
Son’s highest HISCAM, age 23–33	56.24	55.25	55.19	54.46
		(.014)	(.011)	(.075)
Total number	6,537	784	749	117
Females^a^				
Father’s HISCAM birth	52.29	51.96	51.67	52.61
		(.302)	(.051)	(.708)
Father’s highest HISCAM	54.57	53.24	53.63	53.86
		(.000)	(.015)	(.507)
Husband’s highest HISCAM, age 23–33	56.11	55.81	54.86	54.98
		(.488)	(.004)	(.340)
Total number	5,567	649	620	79

^a^Numbers in parentheses are *p* values of two-sample two-tailed *t* tests with nonorphans as the reference group.

The highest father’s HISCAM score ever recorded, however, is related to parental loss: fathers of nonorphans score higher than the fathers of children who had experienced parental death. Interestingly, fathers of male maternal orphans obtained a similar HISCAM score as fathers of nonbereaved children, which indicates that adult fathers improved their social position and experienced intragenerational mobility even when they were confronted with the death of their wife. Accordingly, it is reasonable to take the father’s HISCAM score at birth as a control variable in the multivariate analysis because it is much less related to parental loss. Moreover, the father’s HISCAM score at birth highlights the early-life conditions of the research persons. Finally, concerning the occupational position of sons in young adulthood, the mean values indicate that both paternal death and maternal death are significantly associated with a 1 point lower HISCAM score. Sons-in-law, however, obtain a lower occupational score only if their wife experienced maternal death in childhood.

Because a large proportion of the research persons have no occupation or a husband’s occupation recorded, it is necessary to examine whether their occupational position in childhood differs considerably from that of the studied sample. Comparing average paternal HISCAM scores at birth reveals that children with higher-status background are underrepresented in the study sample (see Table A1, online appendix). Males with no occupational records have significantly higher paternal HISCAM scores at birth compared with both those without an occupation at ages 23–33 and those in the study sample. The same differences in average paternal HISCAM scores at birth are observed for females. Given the positively skewed nature of HISCAM (Lambert et al. 2013), I suspect that these differences are caused by the exceptional position of the elite in Dutch society, as discussed in more detail in the upcoming section on limitations. Furthermore, to overcome this bias in the multivariate analysis, I run robustness checks by assigning the research persons without any occupational entry to their fathers’ highest HISCAM score ever recorded.

### Multivariate Analysis

In the first part of the multivariate analysis, I study the effect of parental death before age 16 on the status attainment of males (Table 3) and females (Table 4). In line with the descriptive findings, Model 1 in Table 3 reveals that parental death among males is associated with a 0.70 lower HISCAM score in young adulthood—a finding that is statistically significant at the 1 % level. The effects of the childhood variables are in the expected directions. The father’s socioeconomic status at his son’s birth largely explains his occupational position in young adulthood. As Fig. 2 suggests, sons born between 1850 and 1879 had the lowest occupational status, but no difference is evident between those born in the subsequent two cohorts. Father’s illiteracy, the total number of siblings, and the number of sibling deaths are associated with lower occupational prestige. The mother’s age at her son’s birth and the religious denomination, however, do not have an impact on son’s career development.

**Table 3:** Ordinary least squares regression with robust standard errors with continuous HISCAM score as dependent variable and parental death as dummy variable, males

	(1)		(2)		(3)	
	Childhood Variables		Orphanhood Variables		Adulthood Variables	
	Coefficient	SE	Coefficient	SE	Coefficient	SE
Orphanhood Status (ref. = no parental death)						
Parental death	–0.703**	0.258	–1.022**	0.332	–0.984**	0.326
Birth Period (ref. = 1900–1922)						
1850–1879	–1.903***	0.290	–1.901***	0.290	–1.762***	0.296
1880–1899	–0.155	0.271	–0.152	0.271	–0.289	0.274
Mother’s Age at Birth (ref. = 25–35)						
<25	–0.233	0.330	–0.230	0.330	–0.246	0.325
>35	–0.249	0.301	–0.229	0.301	–0.206	0.297
Father’s Literacy (ref. = literate)						
Illiterate	–2.575***	0.292	–2.563***	0.293	–2.306***	0.290
Unknown	0.518	0.356	0.528	0.356	–0.190	0.377
Number of Older Brothers	–0.365***	0.0862	–0.367***	0.0863	–0.357***	0.0851
Number of Younger Brothers	–0.474***	0.0758	–0.481***	0.0764	–0.434***	0.0755
Number of Older Sisters	–0.331***	0.0870	–0.328***	0.0871	–0.343***	0.0861
Number of Younger Sisters	–0.315***	0.0783	–0.321***	0.0779	–0.339***	0.0769
Number of Sibling Deaths (ref. = none)						
One	–0.526*	0.255	–0.542*	0.255	–0.649*	0.253
Two or more	–0.895**	0.273	–0.921***	0.274	–1.143***	0.273
Religion (ref. = liberal protestant)						
Catholic	–0.328	0.244	–0.316	0.244	–0.526^†^	0.282
Orthodox protestant	–0.196	0.314	–0.195	0.313	–0.0798	0.323
Unknown/other	–0.0488	0.368	–0.0496	0.369	–0.177	0.369
Father’s HISCAM Score at Birth	0.552***	0.0198	0.552***	0.0198	0.515***	0.020
Presence of Stepparent (ref. = none)						
Stepparent			0.615	0.463	0.732	0.454
Presence of Female Kin (ref. = none present**)**						
Female kin present			0.485	0.611	0.473	0.604
Civil Status (ref. = married)						
Unmarried					0.528*	0.248
Unknown					–0.280	0.432
Provincial Migration and Place of Residence (ref. = no migration/urban)						
No migration/rural					–2.211***	0.257
Migration/urban					1.557**	0.481
Migration/rural					1.803**	0.623
Unknown					–1.655*	0.726
Age at Occupational Record					0.282***	0.0364
Region of Residence	No		No		Yes	
Constant	30.41***	1.102	30.44***	1.101	26.39***	1.495
Number of Observations	8,187		8,187		8,187	
*R* ^2^	.2125		.2128		.2404	
Adjusted *R*^2^	.2109		.2109		.2370	

^†^*p* < .10; *p < .05; **p < .01; ***p < .001

**Table 4:** Ordinary least squares regression with robust standard errors with continuous HISCAM score as dependent variable and parental death as dummy variable, females

	(1)		(2)		(3)	
	Childhood Variables		Orphanhood Variables		Adulthood Variables	
	Coefficient	SE	Coefficient	SE	Coefficient	SE
Orphanhood Status (ref. = no parental death)						
Parental death	–0.503^†^	0.271	–0.646^†^	0.338	-0.525	0.332
Birth Period (ref. = 1900–1922)						
1850–1879	–1.481***	0.295	–1.484***	0.295	–1.381***	0.298
1880–1899	0.252	0.290	0.250	0.290	0.181	0.290
Mother’s Age at Birth (ref. = 25–35)						
<25	0.533	0.360	0.538	0.360	0.526	0.355
>35	–0.856**	0.316	–0.847**	0.316	–0.871**	0.312
Father’s Literacy (ref. = literate)						
Illiterate	–2.174***	0.328	–2.171***	0.327	–1.865***	0.328
Unknown	0.385	0.372	0.383	0.372	–0.565	0.399
Number of Older Brothers	–0.124	0.0878	–0.121	0.0878	–0.102	0.0866
Number of Younger Brothers	–0.332***	0.0805	–0.331***	0.0807	–0.317***	0.0800
Number of Older Sisters	–0.0456	0.0968	–0.0416	0.0969	–0.0780	0.0957
Number of Younger Sisters	–0.387***	0.0805	–0.389***	0.0810	–0.400***	0.0796
Number of Sibling Deaths (ref. = none)						
One	–0.0980	0.284	–0.100	0.284	–0.259	0.279
Two or more	–1.175***	0.290	–1.175***	0.291	–1.365***	0.288
Religion (ref. = liberal protestant)						
Catholic	–0.654*	0.257	–0.653*	0.258	–0.481	0.307
Orthodox protestant	–0.353	0.338	–0.352	0.338	–0.0761	0.344
Unknown/other	0.378	0.425	0.376	0.425	0.484	0.423
Father’s HISCAM Score at Birth	0.562***	0.0222	0.561***	0.0222	0.521***	0.0223
Presence of Stepparent (ref. = none)						
Stepparent			0.0967	0.499	–0.0704	0.492
Presence of Female Kin (ref. = none present**)**						
Female kin present			0.700	0.785	0.627	0.774
Provincial Migration and Place of Residence (ref. = no migration/urban)						
No migration/rural					–2.507***	0.280
Migration/urban					1.316**	0.502
Migration/rural					1.407*	0.652
Unknown					–2.041*	0.802
Husband’s Age at Occupational Record					0.212***	0.0381
Region of Residence	No		No		Yes	
Constant	28.98***	1.209	28.99***	1.209	27.08***	1.636
Number of Observations	6,915		6,915		6,915	
*R* ^2^	.2027		.2028		.2310	
Adjusted *R*^2^	.2007		.2006		.2272	

^†^*p* < .10; *p < .05; **p < .01; ***p < .001

In the second model, I add the presence of stepparents and female kin in the regression. These variables are insignificant, but the negative effect of parental mortality leads to a 1.02 lower HISCAM score. A comparison of the adjusted *R*^2^ of the first and second models shows that taking orphanhood variables into account does not increase the proportion of explained variance. After the adulthood variables are included in Model 3, the negative impact of parental death is hardly affected and remains at a 0.98 lower HISCAM score. The adjusted *R*^2^ increases from 21.1 to 23.7, which means that more variance is explained after adulthood variables are controlled for. Both rural and urban migrants obtained an occupational score much higher than rural and urban stayers. Rural stayers had by far the lowest occupational position. Being older (aged 23–33) is beneficial for one’s own career, which points to intragenerational mobility in young adulthood. Unmarried males had slightly higher occupational status, in line with earlier research on the Netherlands finding a link between low socioeconomic status and earlier transition to first marriage (Engelen and Kok 2002; Rosenbaum-Feldbrügge and Debiasi 2019; Suanet and Bras 2010).

Table 4 shows that female status attainment was much less affected by parental death. In the first model, which only controls for childhood conditions, losing a parent before age 16 is associated with a 0.5 lower HISCAM score, but the finding is significant only at the 10 % level. Including the entry of the stepparents and female kin in the second model does not change the effect substantially, but the relationship becomes insignificant after adulthood confounders are controlled for in Model 3. Regarding the control variables, remarkable similarities and differences between males and females emerge. The father’s socioeconomic position at birth, father’s illiteracy, number of younger siblings, number of sibling deaths, and being born in the first period are associated with lower status attainment for both males and females. Daughter’s occupational position, however, is also reduced by mother’s advanced age at birth and by membership in the Catholic Church. Because Catholic women typically married Catholic men in the period of consideration, this finding indicates weaker economic power of the Catholic minority. The effects of migration and the age of the husband are comparable with those of the male research sample.

To test whether the findings in Table 3 for males are robust, I run three robustness checks depicted in Table A2 in the online appendix. First, the historical social position of self-employed farmers is often unclear, and various occupational schemes place them at different positions in society (Zijdeman 2010). Therefore, I exclude father and son(-in-law) pairs that comprise at least one self-employed farmer. Second, roughly 2,000 males in the nonbereaved control group were not living with both biological parents through age 16. These males are removed from the analysis to compare the bereaved individuals with those from complete families. Third, as described earlier, I assign the highest paternal HISCAM score to those research persons without any occupational entry recorded in the HSN. The robustness checks show that the effect of parental death is not sensitive to these changes and that the results remain statistically significant at the 1 % or 2 % level.

Having shown that parental death is significantly associated with lower status attainment for males but not for females, I next examine more closely the roles of the sex of the deceased parent and the sex of the stepparent, the child’s age at bereavement, and the effect of parental death over time. Table 5 depicts the analysis for males, and Table 6 depicts the analysis for females. Here I control for childhood, orphanhood, and adulthood conditions, because this model explains the largest variance. The effect sizes of the covariates are very similar to those shown in Tables 3 and 4. The first model in Table 5 illustrates that the harmful effect of parental death on son’s status attainment is mainly driven by the loss of the mother, which is associated with a highly significant 1.43 lower HISCAM score in young adulthood. Paternal death, in contrast, is associated with only a nonsignificant 0.68 lower HISCAM score. The joint test performed, however, shows no significant difference between maternal loss and paternal loss (*F* = 1.72; *p* > *F* = .19). Model 1 also shows that the entry of a stepmother attenuates the harmful impact of maternal death, whereas the entry of a stepfather is not associated with a higher status attainment. Differences between stepfathers and stepmothers are also significant at the 5 % level (*F* = 4.32; *p* > *F* = .04).

**Table 5:** Ordinary least squares regression with robust standard errors with continuous HISCAM score as dependent variable and moderator variables, males

	(1)		(2)		(3)	
	Sex of Deceased Parent		Age at Bereavement		Interactions With Time	
	Coefficient	SE	Coefficient	SE	Coefficient	SE
Orphanhood Status (ref. = not orphaned)						
Full orphan	–1.255	0.773			–2.247	2.279
Paternal orphan	–0.675	0.422			0.842	0.939
Maternal orphan	–1.433**	0.459			–0.818	0.753
Orphanhood Status and Age at Bereavement (ref. = not orphaned)						
Paternal orphan, 0–2			–1.116	0.929		
Paternal orphan, 3–6			–0.878	0.812		
Paternal orphan, 7–10			–0.554	0.626		
Paternal orphan, 11–15			–0.607	0.599		
Maternal orphan, 0–2			–2.153*	0.948		
Maternal orphan, 3–6			–1.838*	0.731		
Maternal orphan, 7–10			–2.000**	0.699		
Maternal orphan, 11–15			–0.766	0.585		
Full orphan			–1.372^†^	0.773		
Birth Period (ref. = 1900–1922)						
1850–1879	–1.763***	0.297	–1.755***	0.297	–1.535***	0.327
1880–1899	–0.292	0.274	–0.288	0.274	–0.115	0.302
Interaction With Time						
Full orphan ×1850–1879					1.578	2.464
Full orphan × 1880–1899					0.344	2.608
Full orphan × 1900–1922					ref.	ref.
Paternal orphan × 1850–1879					–2.433*	1.032
Paternal orphan × 1880–1899					–1.411	1.060
Paternal orphan × 1900–1922					ref.	ref.
Maternal orphan × 1850–1879					–0.523	0.944
Maternal orphan × 1880–1899					–1.079	0.897
Maternal orphan × 1900–1922					ref.	ref.
Mother’s Age at Birth (ref. = 25–35)						
<25	–0.242	0.325	–0.237	0.325	–0.219	0.325
>35	–0.230	0.298	–0.251	0.299	–0.242	0.298
Father’s Literacy (ref. = literate)						
Illiterate	–2.317***	0.291	–2.315***	0.291	–2.332***	0.291
Unknown	–0.196	0.377	–0.196	0.377	–0.213	0.375
Number of Older Brothers	–0.354***	0.0851	–0.353***	0.0850	–0.348***	0.0853
Number of Younger Brothers	–0.437***	0.0756	–0.444***	0.0757	–0.440***	0.0755
Number of Older Sisters	–0.339***	0.0863	–0.334***	0.0866	–0.338***	0.0864
Number of Younger Sisters	–0.340***	0.0771	–0.347***	0.0777	–0.340***	0.0771
Number of Sibling Deaths (ref. = none)						
One	–0.642*	0.253	–0.646*	0.253	–0.654*	0.254
Two or more	–1.136***	0.273	–1.143***	0.274	–1.151***	0.273
Religion (ref. = liberal protestant)						
Catholic	–0.524^†^	0.282	–0.524^†^	0.282	–0.532^†^	0.282
Orthodox protestant	–0.083	0.323	–0.0882	0.324	–0.106	0.322
Unknown/Other	–0.171	0.369	–0.181	0.369	–0.188	0.369
Father’s HISCAM Score at Birth	0.515***	0.0201	0.515***	0.020	0.515***	0.020
Presence of Stepparent (ref. = none)						
Stepfather	–0.328	0.659	–0.200	0.681	–0.301	0.658
Stepmother	1.526*	0.611	1.799**	0.627	1.547*	0.617
Presence of Female Kin (ref. = none present)						
Female kin present	0.427	0.606	0.587	0.608	0.480	0.611
Civil Status (ref. = married)						
Unmarried	0.530*	0.248	0.534*	0.248	0.532*	0.248
Unknown	–0.286	0.433	–0.295	0.433	–0.298	0.432
Provincial Migration and Place of Residence (ref. = no migration/urban)						
No migration/rural	–2.198***	0.257	–2.190***	0.257	–2.199***	0.256
Migration/urban	1.574**	0.481	1.572**	0.481	1.563**	0.481
Migration/rural	1.816**	0.623	1.821**	0.624	1.832**	0.623
Unknown	–1.646*	0.727	–1.618*	0.727	–1.637*	0.727
Age at Occupational Record	0.283***	0.0365	0.284***	0.0365	0.283***	0.0365
Region of Residence	Yes		Yes		Yes	
Constant	26.39***	1.496	26.35***	1.496	26.23***	1.497
Number of Observations	8,187		8,187		8,187	
*R* ^2^	.2407		.2410		.2415	
Adjusted *R*^2^	.2371		.2368		.2374	

^†^*p* < .10; *p < .05; ** *p* < .01; ***p < .001

**Table 6:** OLS regression with robust standard errors with continuous HISCAM score as dependent variable and moderator variables, females

	(1)		(2)		(3)	
	Sex of Deceased Parent		Age at Bereavement		Interactions With Time	
	Coefficient	SE	Coefficient	SE	Coefficient	SE
Orphanhood Status (ref. = no parental death)						
Full orphan	–1.418	0.996			–1.831	1.690
Paternal orphan	–0.050	0.421			–0.773	0.873
Maternal orphan	–1.173*	0.498			–1.612*	0.739
Orphanhood Status and Age at Bereavement (ref. = no parental death)						
Paternal orphan, 0–2			0.491	1.011		
Paternal orphan, 3–6			–1.157^†^	0.691		
Paternal orphan, 7–10			0.321	0.760		
Paternal orphan, 11–15			0.0455	0.595		
Maternal orphan, 0–2			–1.745	1.115		
Maternal orphan, 3–6			–1.551^†^	0.912		
Maternal orphan, 7–10			–1.821**	0.688		
Maternal orphan, 11–15			–0.373	0.625		
Full orphan			–1.491	1.000		
Birth Period (ref. = 1900–1922)						
1850–1879	–1.381***	0.298	–1.373***	0.298	–1.452***	0.331
1880–1899	0.181	0.290	0.203	0.291	0.0158	0.322
Interaction With Time						
Full orphan × 1850–1879					0.548	2.078
Full orphan × 1880–1899					0.524	2.637
Full orphan × 1900–1922					ref.	ref.
Paternal orphan × 1850–1879					0.599	1.023
Paternal orphan × 1880–1899					1.222	1.020
Paternal orphan × 1900–1922					ref.	ref.
Maternal orphan × 1850–1879					0.394	0.908
Maternal orphan × 1880–1899					0.742	0.921
Maternal orphan × 1900–1922					ref.	ref.
Mother’s Age at Birth (ref. = 25–35)						
<25	0.549	0.356	0.560	0.356	0.540	0.356
>35	–0.895**	0.312	–0.904**	0.313	–0.891**	0.312
Father’s Literacy (ref. = literate)						
Illiterate	–1.878***	0.326	–1.869***	0.325	–1.887***	0.327
Unknown	–0.575	0.399	–0.581	0.399	–0.572	0.400
Number of Older Brothers	–0.0985	0.0865	–0.0984	0.0865	–0.100	0.0866
Number of Younger Brothers	–0.322***	0.0801	–0.327***	0.0801	–0.321***	0.0802
Number of Older Sisters	–0.0697	0.0958	–0.0657	0.0959	–0.0694	0.0960
Number of Younger Sisters	–0.398***	0.0797	–0.399***	0.0799	–0.396***	0.0799
Number of Sibling Deaths (ref. = none)						
One	–0.253	0.279	–0.238	0.279	–0.259	0.278
Two or more	–1.358***	0.288	–1.352***	0.289	–1.362***	0.289
Religion (ref. = liberal protestant)						
Catholic	–0.486	0.307	–0.485	0.307	–0.492	0.307
Orthodox protestant	–0.0649	0.344	–0.0644	0.344	–0.0635	0.344
Unknown/other	0.491	0.424	0.489	0.424	0.490	0.424
Father’s HISCAM Score at Birth	0.521***	0.0223	0.521***	0.0223	0.521***	0.0223
Presence of Stepparent (ref. = none)						
Stepfather	–1.131	0.766	–1.006	0.796	–1.165	0.764
Stepmother	0.863	0.664	0.996	0.723	0.841	0.671
Presence of Female Kin (ref. = none present)						
Female kin present	0.654	0.777	0.793	0.782	0.645	0.780
Provincial Migration and Place of Residence (ref. = no migration/urban)						
No migration/rural	–2.498***	0.281	–2.508***	0.281	–2.502***	0.281
Migration/urban	1.299**	0.502	1.287*	0.501	1.297**	0.502
Migration/rural	1.426*	0.653	1.419*	0.654	1.413*	0.653
Unknown	–1.998*	0.804	–1.978*	0.806	–2.001*	0.804
Age at Occupational Record	0.211***	0.0380	0.212***	0.0380	0.210***	0.0380
Region of Residence	Yes		Yes		Yes	
Constant	27.07***	1.635	27.07***	1.635	27.18***	1.639
Number of Observations	6,915		6,915		6,915	
*R* ^2^	.2315		.2321		.2317	
Adjusted *R*^2^	.2273		.2273		.2269	

^†^*p* < .10; *p < .05; ** *p* < .01; ***p < .001

Model 2 in Table 5 analyzes the impact of age at parental death. Maternal death in the first years of life (until age 10) is associated with a much lower occupational status in young adulthood, whereas maternal loss between ages 11 and 15 is not associated with a statistically significant decline in occupational status. However, joint tests show no significant differences among any of the four age categories, and the same goes for the father’s death (results not shown). Finally, Model 3 examines interaction effects between parental death during childhood and the birth period. When compared with the last birth cohort, the model shows that the negative effect of paternal death during childhood is mainly driven by the research persons born between 1850 and 1879. According to the joint test performed, however, the first and the second period do not differ significantly (*F* = 1.90; *p* > *F* = .17). No interaction effects for maternal orphans and full orphans are found.

Table 6 shows the same multivariate analysis for females and husband’s HISCAM score in young adulthood. As in the case of sons, daughter’s occupational position is negatively affected by maternal death (–1.17) but not by paternal death. Joint tests additionally show that the differences between maternal death and paternal death are significant at the 10 % level (*F* = 3.27; *p* > *F* = .07). In contrast to the findings for sons, the presence of a stepmother does not have a positive impact on daughter’s status attainment. Moreover, the age at parental death does not reveal systematic results (Model 2), and no differences over time are found (Model 3) among females.

The same robustness checks as before are applied for the male and the female sample (results available upon request). The results remain robust and statistically significant. Only the period interaction in the male sample becomes insignificant after the exclusion of farmers, as shown in Table A3 in the online appendix.

## Concluding Discussion

This study examines the relationship between parental death and occupational position in adulthood, a largely neglected topic in historical and contemporary social research. For this purpose, I analyze life course data of more than 8,000 males and nearly 7,000 females born in the Netherlands between 1850 and 1922, a period of great structural labor market transformations. Roughly 20 % of these individuals had lost a parent before age 16. The results suggest that parental death is significantly associated with a lower HISCAM score for males, also after orphanhood and adulthood conditions are controlled for. This is in line with H1a and H1b. In contrast to these hypotheses, however, I do not find strong evidence that parental loss also decreases the occupational position of the husbands of females in the sample. Regarding the other childhood conditions, the results show that having an illiterate father and having experienced the loss of two or more siblings are strongly associated with a substantially lower status attainment in adulthood. A high number of sibling deaths may point to low parenting quality and direct exposure to infections generally associated with scarred health in later life (van Dijk et al. 2018). It is therefore reasonable to treat the exposure to sibling mortality within the family as a proxy for adverse childhood conditions.

The second part of the multivariate analysis reveals that the negative effect on socioeconomic status is driven mainly by maternal loss for both males and females. For instance, maternal death is associated with a highly significant 1.43 lower HISCAM score among sons, whereas paternal death is associated with an insignificant 0.68 lower score. The difference between the effect of maternal and paternal death for females is also significant at the 10 % level. In contrast to H2, the results therefore indicate that maternal death appears to be more detrimental for one’s social position in later life than paternal death. Maternal death in childhood not only has dramatic short-term consequences on survival chances in infancy and childhood (Sear and Mace 2008) but also results in serious long-term socioeconomic disadvantages. The findings therefore point to the crucial importance of maternal care and parenting quality on outcomes in later life. Surprisingly, the loss of income and material resources, which is linked in my theoretical framework to paternal death, does not appear to be harmful. Mothers were apparently better able than fathers to cope with the loss of their partner and to provide for the basic needs of their children. Possibly, the support offered by institutionalized poor relief, specifically to widows, also contributed to the mothers’ resilience (Schmidt 2007).

Even though maternal death early in life is associated with a larger negative effect size and maternal death in adolescence is not significantly associated with a low HISCAM score, no substantial evidence is found for the hypothesis that bereavement in early life is significantly worse than bereavement in adolescence (H3). The conclusion that maternal care is crucial, however, is supported by the large positive effect on son’s occupational position associated with the entry of a stepmother. In contrast to H4, the entry of a stepfather did not enhance the boys’ and girls’ labor market performance. Father’s remarriage was apparently a successful strategy to provide for childcare services and to stabilize the household. The positive stepmother effect, however, might also reflect the unobserved quality of the father seeking to remarry, which would also contribute to the male offspring’s higher social attainment. Accordingly, the effect might also be attributed to social selection rather than a causal mechanism.

The final hypothesis (H5) assumes that parental death was more detrimental in the beginning than in the end of the research period given that the end of the nineteenth and start of the twentieth century marked the onset of a period of great structural labor market transformations and increasing opportunities for upward mobility. Absolutely no evidence for the hypothesis is found with regard to maternal death, which appears to be detrimental throughout the entire period. Once again, this indicates that maternal care is crucial for later-life outcomes independent of external circumstances for both sons and daughters. Interaction effects with time, however, indicate that the loss of the father was more harmful in the beginning than in the end of the research period. The rapid increase in the demand for educated nonmanual workers in the beginning of the twentieth century (van Zanden and van Riel 2004) led to a decrease in direct inheritance of occupation from father to son (Knigge et al. 2014; Schulz et al. 2015; Treiman 1970). As a result, sons became less dependent on their father’s occupational background and their father’s survival. The interaction effect, however, is sensitive to the exclusion of farmers. This finding should therefore be taken with caution.

Although I run several robustness checks to reinforce the conclusions, three limitations of the study are noteworthy. First, information about the parents’ cause of death is not available in the HSN. This is problematic given the findings of Rostila and colleagues (Rostila et al. 2016) that young Swedish adults who had lost a parent due to an external cause of death had an increased risk of hospitalization as a result of a self-inflicted injury compared with their counterparts who had lost a parent due to natural causes. Accordingly, death from external causes could also have more detrimental effects on occupational status in young adulthood because it is to a larger extent associated with posttraumatic stress (Merlevede et al. 2004). Even though the HSN includes no individual-level information on parental cause of death, aggregated cause-of-death data in the Netherlands are available. The aggregated data show that only 2 % to 3 % of total mortality was registered as external causes and suicides in both 1880 and 1917, and that most adults in these years died from infectious diseases (Wolleswinkel-van den Bosch et al. 1998). Therefore, the large majority of children can be assumed to have lost their parents to natural causes of death. External causes did not play an important role, at least when compared with the Swedish sample, in which 40 % of the paternal deaths and 25 % of the maternal deaths were due to external causes and/or substance abuse.

The second limitation is the underrepresentation of children with higher-status background in the sample. As shown in the descriptive statistics, those with no occupation recorded in the registers and certificates were more likely to be born to fathers with higher HISCAM scores. This was observed both for children who experienced parental death and those who did not. Earlier research on the Netherlands has shown that sons and daughters from the elite classes had the highest proportions of permanent celibacy and that children born to casual and unskilled workers were much more likely to marry (Engelen and Kok 2002). This explains why the information derived from the marriage certificates is biased toward lower social classes. There is also some indication that the members of the elite were reluctant to register occupations in the population registers or did not even pursue a profession given that they mainly managed their family’s property (Furnée 2012:81–84, 749). Nevertheless, I do not believe that the underrepresentation of individuals from the highest social classes calls the general findings of the study into question because the underrepresentation occurred to both those who experienced parental death and those who did not. This conclusion is supported by the robustness checks, which assigned the highest paternal occupation to the individuals without any occupational record.

Finally, results show that daughter’s socioeconomic position is generally much less affected by parental death than son’s socioeconomic position. This suggests that daughters are more robust when it comes to the negative long-term consequences of parental death in childhood. However, this finding might also be attributed to measurement problems in this study: daughter’s socioeconomic position is indirectly estimated by taking husband’s occupation into account, and I believe that this imperfect measure only partly covers the effect of parental death. Accordingly, historical data on female occupational position are generally disadvantaged compared with data on contemporary populations, which typically contain information about female occupations. Therefore, I suggest exploiting contemporary survey and register data to carefully examine whether daughters are less sensitive to parental death in childhood than sons.

To conclude, recent research on historical social mobility in the Netherlands has tended to examine the impact of industrialization on occupational status attainment without taking individual childhood conditions and family crises into account (Knigge 2016; Knigge et al. 2014; Zijdeman 2010). The results of this article, however, suggest that the history of social mobility is both a history of the family and a history of modernization and structural labor market transformations (Kaelble 1986). Analyzing the development of social mobility over time without taking into account childhood conditions (such as paternal literacy) as well as family crises (such as parental and sibling mortality) ignores important explanatory factors for status attainment. This study therefore emphasizes the complex interaction between individual life courses and the surrounding social, economic, and institutional developments (Kok 2007). Additionally, studies on parental death in contemporary societies rarely differentiate between maternal and paternal death (e.g., Amato and Anthony 2014; Björkenstam et al. 2016; Feigelman et al. 2017). My theoretical framework and results, however, suggest that maternal and paternal death operate through different mechanisms and should be considered separately. Finally, future research should further disentangle the important role of moderating factors, such as the sex of the deceased parent, the presence of stepparents, and age at parental death, in order to identify the most vulnerable children growing up in today’s societies.

## Electronic supplementary material

ESM 1 (PDF 581 kb)

## Footnotes

1 The turn of the century witnessed the emergence of widows’ funds and of social security to improve the living standards of vulnerable citizens (van Loo 1981; van Poppel 1995). Nevertheless, poor relief and social security in the Netherlands were not sufficient to compensate for the loss of income caused by the death of the male household head.

2 Occupations were coded into HISCO, HISCAM, and HISCLASS using a data set Mandemakers et al. (2013) provided for HSN.

3 I use STATA’s *vce* (robust) option to calculate the robust standard errors.

4 This finding is in line with earlier research that did not find evidence for a link between socioeconomic position and adult and later-life mortality in the nineteenth century. See Bengtsson and van Poppel (2011) and Mourits (2017) for recent overviews.
