# Supplementary material for: The Impact of Parental Death in Childhood on Sons’ and Daughters’ Status Attainment in Young Adulthood in the Netherlands, 1850–1952
Source: Demography. 2019 Aug 16;56(5):1827–54. doi: 10.1007/s13524-019-00808-z (PMC6797636; doi:10.1007/s13524-019-00808-z)
Supplement: Supplementary file 1 — (PDF 581 kb) [file 13524_2019_808_MOESM1_ESM.pdf]

ONLINE APPENDIX

Table A1: Father's mean HISCAM scores at birth to compare the sample population with those without occupational information

	**Study sample**	**No occupation between the ages of 23 and 33**	**No occupation at all**
**Male research persons**			
Father's HISCAM at birth	52.46	52.80<br>(0.091)	54.97<br>(0.000)
Total numbers	8,187	1,946	1,857
**Female research persons**			
Father's HISCAM at birth	52.21	52.48<br>(0.210)	54.87<br>(0.000)
Total numbers	6,915	1,442	3,698

Table A2: OLS regression with robust standard errors with continuous HISCAM score as dependent variable and parental death as dummy variable, male research persons, robustness checks[a]

	(1)[b] Childhood variables		(2)[c] Orphanhood variables		(3)[d] Adulthood variables	
	Coefficient	SE	Coefficient	SE	Coefficient	SE
**(I) Farmers excluded**						
**Orphanhood status**						
Parental death	-0.803**	0.294	-1.336***	0.379	-1.120**	0.355
No parental death	ref	ref	ref	ref	ref	ref
Observations	6,700		6,700		6,700	
R-squared	0.235		0.236		0.259	
**(II) Non-bereaved sons who were not living with both biological parents excluded**						
**Orphanhood status**						
Parental death	-0.773**	0.270	-1.048**	0.328	-1.040**	0.323
No parental death	ref	ref	ref	ref	ref	ref
Observations	6,103		6,103		6,103	
R-squared	0.209		0.210		0.241	
**(III) Highest paternal HISCAM score assigned to research persons without any occupational record**						
**Orphanhood status**						
Parental death	-0.554*	0.225	-0.789**	0.275	-0.731**	0.272
No parental death	ref	ref	ref	ref	ref	ref
Observations	10,044		10,044		10,044	
R-squared	0.313		0.313		0.334	

† p<0.1, * p<0.05, ** p<0.01, *** p<0.001

[a] Full tables are available upon request.

[b] Controlled for period of birth, mother's age at birth, father's literacy, number of younger and older brothers and sisters, number of sibling deaths, religious denomination, and paternal HISCAM score at birth.

[c] Controlled for period of birth, mother's age at birth, father's literacy, number of younger and older brothers and sisters, number of sibling deaths, religious denomination, paternal HISCAM score at birth, presence of step-parents, and presence of female kin.

[d] Controlled for period of birth, mother's age at birth, father's literacy, number of younger and older brothers and sisters, number of sibling deaths, religious denomination, paternal HISCAM score at birth, presence of step-parents, presence of female kin, civil status, provincial migration and place of residence, age at occupational record, and region of residence. Adulthood variables of male individuals without occupational entry are measured at age 27, the rounded sample average.

Table A3: OLS regression with robust standard errors with continuous HISCAM score as dependent variable and moderator variables, male research persons, farmers excluded[a]

	(1) Sex of deceased parent		(2) Age at bereavement		(3) Interactions with time	
	Coefficient	SE	Coefficient	SE	Coefficient	SE
**Farmers excluded**						
**Orphanhood status**						
Full orphan	-0.900	0.988			-2.199	3.197
Paternal orphan	-0.806†	0.461			-0.336	0.980
Maternal orphan	-1.758***	0.501			-0.700	0.849
No parental death	ref	ref			ref	ref
**Orphanhood status and age at bereavement**						
Paternal orphan, 0-2			-1.459	0.919		
Paternal orphan, 3-6			-0.944	0.980		
Paternal orphan, 7-10			-0.659	0.641		
Paternal orphan, 11-15			-0.657	0.693		
Maternal orphan, 0-2			-2.630**	0.915		
Maternal orphan, 3-6			-1.768*	0.827		
Maternal orphan, 7-10			-2.406**	0.801		
Maternal orphan, 11-15			-1.106†	0.655		
Full orphan			-0.970	0.989		
No parental death			ref	ref		
**Interaction with time**						
Full orphan X 1850-1879					1.930	3.406
Full orphan X 1880-1899					0.638	3.621
Full orphan X 1900-1922					ref	ref
Paternal orphan X 1850-1879					-1.267	1.098
Paternal orphan X 1880-1899					0.011	1.145
Paternal orphan X 1900-1922					ref	ref
Maternal orphan X 1850-1879					-1.388	1.072
Maternal orphan X 1880-1899					-1.486	1.024
Maternal orphan X 1900-1922					ref	ref
**Presence of step-parent**						
Step-father	0.0417	0.762	0.201	0.788	0.0735	0.762
Step-mother	1.975**	0.686	2.210**	0.693	2.044**	0.696
No step-parent	ref	ref	ref	ref	ref	ref
Observations	6,700		6,700		6,700	
R-squared	0.2595		0.2598		0.2601	

† p<0.1, * p<0.05, ** p<0.01, *** p<0.001

[a] Controlled for period of birth, mother's age at birth, father's literacy, number of younger and older brothers and sisters, number of sibling deaths, religious denomination, paternal HISCAM score at birth, presence of step-parents, presence of female kin, civil status, provincial migration and place of residence, age at occupational record, and region of residence. Full table is available upon request.

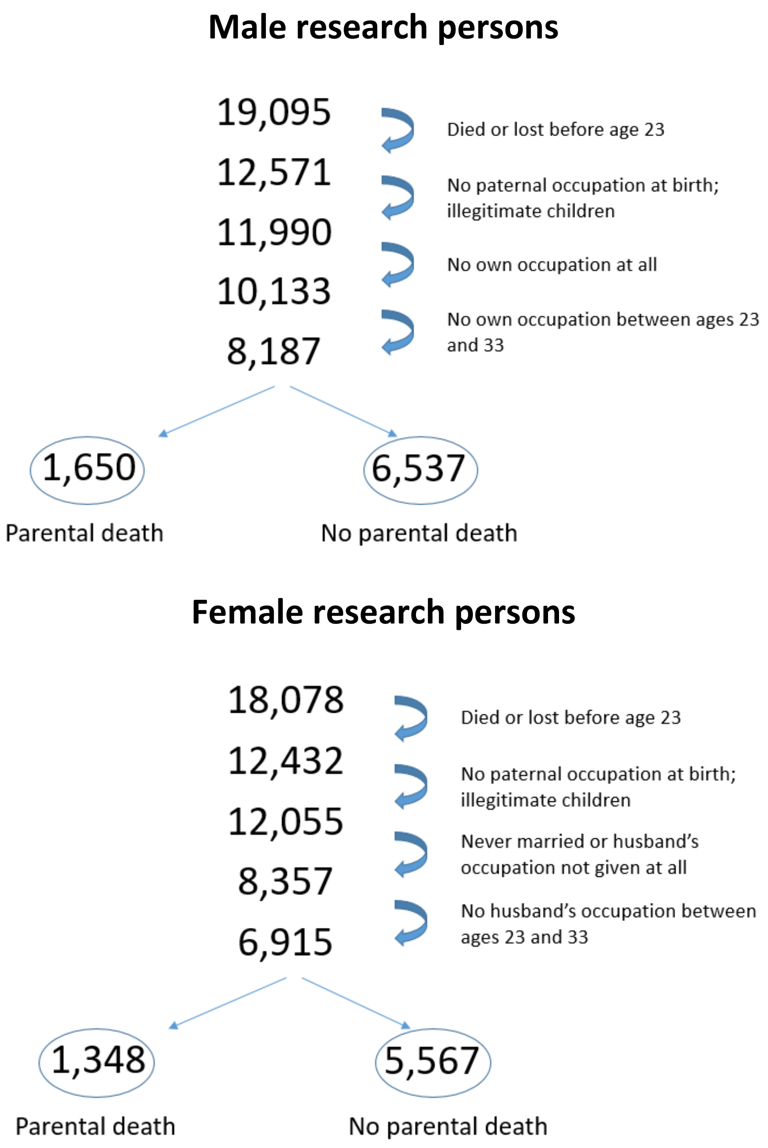

Fig. A1 Overview of sample selection process
